# Stimulus size modulates idiosyncratic neural face identity discrimination

**DOI:** 10.1167/jov.22.13.9

**Published:** 2022-12-29

**Authors:** Lisa Stacchi, Roberto Caldara

**Affiliations:** 1 Eye and Brain Mapping Laboratory (iBMLab), Department of Psychology, University of Fribourg, Fribourg, Switzerland

**Keywords:** idiosyncrasies, face discrimination, stimulus size, viewing positions, EEG-FPVS

## Abstract

Humans show individual differences in neural facial identity discrimination (FID) responses across viewing positions. Critically, these variations have been shown to be reliable over time and to directly relate to observers' idiosyncratic preferences in facial information sampling. This functional signature in facial identity processing might relate to observer-specific diagnostic information processing. Although these individual differences are a valuable source of information for interpreting data, they can also be difficult to isolate when it is not possible to test many conditions. To address this potential issue, we explored whether reducing stimulus size would help decrease these interindividual variations in neural FID. We manipulated the size of face stimuli (covering 3°, 5°, 6.7°, 8.5°, and 12° of visual angle), as well as the fixation location (left eye, right eye, below the nasion, nose, and mouth) while recording electrophysiological responses. Same identity faces were presented with a base frequency of 6 Hz. Different identity faces were periodically inserted within this sequence to trigger an objective index of neural FID. Our data show robust and consistent individual differences in neural face identity discrimination across viewing positions for all face sizes. Nevertheless, FID was optimal for a larger number of observers when faces subtended 6.7° of visual angle and fixation was below the nasion. This condition is the most suited to reduce natural interindividual variations in neural FID patterns, defining an important benchmark to measure neural FID when it is not possible to assess and control for observers' idiosyncrasies.

## Introduction

An increasingly large body of work is providing evidence of functionally meaningful individual differences in face processing. Eye movement studies have reported significant differences across cultures during face recognition (e.g., Western Caucasians vs. East Asians; e.g., Blais et al., 2008; Kelly et al., 2010; Miellet et al., 2012; for a review, see Caldara, 2017). Notably, variations have also been reported between individuals from the same cultural background during face identification, suggesting that observers have idiosyncratic visual sampling strategies that are not well represented by group averages (Mehoudar et al., 2014; Walker-Smith et al., 1977). These idiosyncrasies have been shown to be reliable over a period of 18 months (Mehoudar et al., 2014), across settings (i.e., lab vs. real world; Peterson et al., 2016), and to be sufficiently distinct to allow algorithms to identify an observer from their scanning paths (Kanan et al., 2015). Crucially, such individual differences in sampling strategies do not appear to correlate with behavioral performance in identity-related tests. That is, individuals exhibiting different fixation biases can attain similar performance levels (Arizpe et al., 2017, Blais et al., 2008). However, forcing observers to fixate away from their naturally preferred viewing position (VP) is detrimental to their face identification performance (Peterson & Eckstein, 2013), suggesting that these idiosyncrasies are functionally meaningful.

Recently, individual differences in face discrimination have also been documented at the neural level by means of the fast periodic visual stimulation (FPVS) electrophysiological approach (Stacchi, Liu-Shuang, et al., 2019). FPVS consists in presenting faces of varying identities embedded at periodic intervals in a stream of same-identity faces displayed at a particular base frequency (e.g., AAAAAABAAAAAACAAAAAAD . . .). A neural response at the same frequency of identity changes provides an index of the ability of the neural system to discriminate between identities (Liu-Shuang et al., 2014; Rossion et al., 2020). Importantly, studies have shown that the amplitude of the response triggered by FPVS is positively correlated with behavioral performance for face recognition tests (Cambridge Face Memory Test, Xu et al., 2017; Benton Face Facial Recognition Test, Dzhelyova et al., 2020) and a FPVS-like identity discrimination task (Retter et al., 2021). These results support the notion that the strength of the neural FID as recorded using the FPVS–electroencephalogram (EEG) approach is a useful tool to index face identity processing.

Recent findings also show that presenting faces at diverse facial viewing positions (e.g., left eye, right eye, etc.) leads to significant variations across participants in terms of neural amplitude patterns and topographies (Stacchi, Liu-Shuang, et al., 2019; see Xu et al., 2017, for similar individual differences during fixation of a central viewing position [VP] just below the nasion). VP-dependent neural responses differed across individuals, with some participants exhibiting larger responses while fixating, for example, the left eye and others during fixation of the nose (Stacchi, Ramon, et al., 2019). Interestingly, the more a face region was fixated by an observer during natural viewing, the more likely this region was to trigger a strong neural facial identity discrimination (FID) response in the EEG-FPVS. Furthermore, these robust idiosyncrasies across VPs were reliable over a period of 6 months (Stacchi, Ramon, et al., 2019; see Dzhelyova et al., 2019, for 2-month reliability of the FPVS response at the central VP). Altogether, these findings suggest that individual differences recorded through EEG-FPVS can reflect genuine and functionally meaningful variations in face discrimination.

These reliable and meaningful variations provide a unique tool for researchers to investigate the relationship between different processes and how the visual system can flexibly adapt to individual's idiosyncrasies to achieve the same goal of facial identity processing (Stacchi, Liu-Shuang, et al., 2019; Stacchi, Ramon, 2019, for a discussion). These findings are conceptually in stark contrast with the traditional experimental methodology of enforcing fixation on a predefined viewing position that is the same across all subjects. This approach stems from the need to standardize the visual input; if the fixation location is the same, each visual system will receive the same information, and therefore it should be possible to compare their response. This might be true under some research questions, but it is not a cure-all. For example, it would have severe implications if a researcher was to assess neural FID at a standardized location and use this to measure a representative index of individuals’ face system abilities. Within this context, the visual input would be the same, but how well a given system could operate on such information would not be a given, and it would result in some systems facing greater challenges than others because they were submitted with a potentially nonoptimal input. The consequence would be that a subset of the tested observers could exhibit a neural response that could be categorized as weak but that would not be a realistic index of their neural FID. This misrepresentation could in in turn lead to misleading and noisy data interpretation.

To circumvent this issue, researchers should ideally test each subject across multiple viewing positions, which would, however, considerably increase testing duration. This is not always a realistic goal, neither with traditional event-related potentials (ERPs) nor with the FPVS paradigm, which requires significantly less time than the former to extract high signal-to-noise ratio (SNR) responses. For instance, young or clinical populations might be unable to undergo long testing sessions. Additionally, the need to test multiple viewing positions would result in less time available to test other experimental manipulations.

Alternatively, researchers could also first record the oculomotor behavior of observers to establish their preferred viewing position and only subsequently proceed to the EEG recording. However, this approach would require at least two sessions and two different techniques, engendering heavy experimental constraints.

To overcome these issues, the current study aimed to determine if it is possible to reduce the extent of individual differences across VPs as expressed during neural face identity discrimination. Essentially, altering the viewing position of a face induces a change in the information input to the visual system. More specifically, in terms of information intake, the face region that is fixated is sampled at high resolution as the fovea is the portion of the retina with maximal visual acuity, while the facial information surrounding the point of foveation is sampled at a lower resolution. This is highly relevant when stimuli are relatively large and only a small portion of a face can be sampled within the fovea and a single fixation. In our previous studies, we used relatively large face stimuli (i.e., ∼11° of vertical visual angle; Stacchi, Liu-Shuang, et al., 2019; Stacchi, Ramon, et al., 2019). We thus expect that when stimuli are smaller and most of the facial features can be sampled within one fixation, the viewing position will play a minor role in information gathering. If this prediction is correct, then adjusting stimulus size could help to reduce individual differences in neural responses for FID across VPs and therefore make participants more comparable within the group.

To this aim, we thus recorded EEG signals of observers while face images were presented through fast periodic visual stimulation to trigger neural FID. Experimental conditions varied along two dimensions, each with five levels: viewing position (left eye, right eye, nose, mouth, and just below the nasion) and size (3°, 5°, 6.7°, 8.5°, and 12° of vertical visual angle). We expect stimulus size to impact both the response amplitude, independently of VPs, and response patterns across VPs. First, we expect identity processing to be more negatively impacted for small faces than for larger ones (Yang et al., 2014). This might occur because when stimuli are too small, it might become more difficult to rapidly and efficiently extract the fine-grained information necessary to successfully discriminate between face identities. This should lead to decreased and nonsignificant FID responses. To probe this hypothesis, our analysis will first focus on determining the minimum size at which we can reliably obtain valid responses. Only once those conditions are identified will we assess the impact of VP on response patterns.

Then, for the significant neural FID responses, we hypothesize that for smaller face sizes, the point of fixation will no longer be relevant because almost all of the information could be sampled by fixating any facial region. This would result in participants no longer showing preferences for specific VPs. Therefore, in this scenario, the arbitrary choice of a fixed VP when measuring neural FID would not be problematic. Alternatively, it is possible that as stimuli become smaller, observers’ VP-related biases will not necessarily be reduced but will nonetheless converge toward the same region. When the whole face can be sampled through one fixation only, observers could benefit more from a central viewing position (i.e., just below the nasion; see Blais et al., 2008; Miellet et al., 2013), which allows for the simultaneous sampling of a larger portion of the face foveally.

Regardless of these hypotheses, identifying an experimental condition that systematically triggers significant neural FID and simultaneously reduces individual variations in response patterns across VPs would represent a significant methodological advance. It would also increase the already impressive efficiency of the FPVS technique in establishing a representative index of neural FID in only a few minutes.

## Materials and methods

### Participants

We tested a sample of 30 young Caucasian adults, mainly undergraduate students at the University of Fribourg (7 males, 2 left-handed, mean age: 22.4 ± 2.5, range: 18–30). Participants had normal or corrected-to-normal vision, and none had reported to have a history of psychiatric or neurological disorders. They all provided written consent prior to the experiment. The study was approved by the local ethics committee and conformed to the Declaration of Helsinki.

### Procedure

#### Stimuli and procedure

Stimuli consisted in images of 50 face identities (25 females), all displaying a neutral expression. These stimuli have been used in previous EEG-FPVS studies (e.g., Liu-Shuang et al., 2014; Stacchi, Liu-Shuang, et al., 2019; Stacchi, Ramon, et al., 2019; first presented in a study by Laguesse et al., 2012). Faces were all full-front, colored, cropped to remove external facial features, and embedded in a gray background. Stimuli were presented on a VIEWPixx/3D monitor (1920 × 1080-pixel resolution, 120 Hz refresh rate) by means of MATLAB 2016B (PsychToolbox and a custom graphics toolbox). Within each 62-s trial, a stream of faces was presented through sinusoidal contrast modulation at a base frequency of 6 Hz; hence, each stimulus lasted 0.166 ms. This frequency was used because it has been shown to be the most optimal to trigger neural FID in the context of an FPVS paradigm (Alonso-Prieto et al., 2013; Retter et al., 2021).

**Figure 1.**
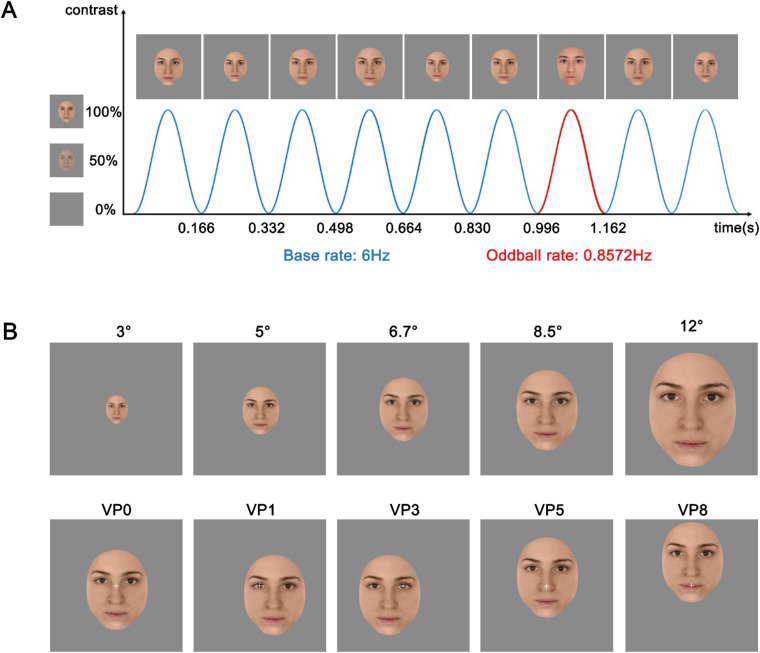
Schematic illustration of the paradigm and the experimental conditions. (A) Faces were presented using fast periodic visual stimulation and a sinusoidal contrast. (B) Conditions varied in terms of stimulus size (top row) and viewing position (bottom row). The face images depicted in this figure were created by the authors, were not used in the study, and are for illustration purposes only. Permission to use these identities has been granted to the authors.

Each sequence consisted in one randomly selected base face that was repeated throughout the whole trial and different randomly selected oddball identities (different from the base identity) interleaved periodically every seventh base face (i.e., 6 Hz/7 = 0.85716 Hz) (Figure 1A). Within each trial, face size randomly varied between 90% and 110% in order to minimize pixel overlap. This 20% variation has been shown to be sufficient to reduce low-level adaptation (Dzhelyova & Rossion, 2014).

In order to maintain attention and fixation, participants were instructed to monitor a fixation cross presented in the middle of the screen and overlapping the images stream. The cross changed color randomly 8 to 10 times each trial, and subjects had to report by button press the occurrence of such change. At this task, participants performed nearly at ceiling (*M* = 0.92, *SD* = 0.12).

**Figure 2.**
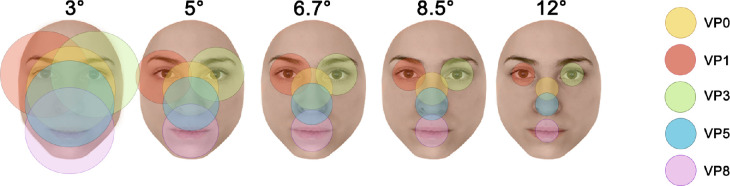
Schematic illustration of a 2° foveal visual field depending on stimulus size and VP. To allow for an easier comparison of the foveal coverage across conditions, the foveal visual field was resized accordingly. The face images depicted in this figure were created by the authors, were not used in the study, and are for illustration purposes only. Permission to use this identity has been granted to the authors.

Trials varied along two dimensions: stimulus size and VP. Size was parametrically modulated across five steps (3°, 5°, 6.7°, 8.5°, and 12° of vertical visual angle). These sizes were chosen in order to not overlap despite the within-trial 20% variation and were obtained by rescaling the stimuli. We varied the VP by arranging faces so that one out of five facial regions were aligned with the center of the screen and hence the fixation cross. VP consisted of left and right eyes (VP1 and VP3, respectively), nose (VP5), mouth (VP8), and a region slightly below the nasion (VP0; Figure 1B and Figure 2). The last VP was selected to include the fixation location typically tested with this paradigm while the remaining VPs were selected to induce fixation on the main facial features.

Each combination of size and VP was presented twice, once with male faces and once with female faces, resulting in a total of 50 trials (5 sizes × 5 VPs × 2 repetitions). Trials with evident motion-related noise contamination were repeated at the end of the experiment (*M* = 3). Including numerous breaks taken to ensure subjects’ attentive state, testing lasted approximately 1.5 h.

#### EEG acquisition

EEG was acquired by means of BioSemi (Amsterdam, Netherlands) ActiView software with a Biosemi Active-Two amplifier system and 128 Ag-AgCl Active electrodes. Offset was lowered and maintained below 25 mV relative to the common mode sense (CMS) and driven right leg (DRL) by slightly abrading the scalp and adding saline gel. The signal was digitalized at a sampling rate of 1024 Hz and online bandpass filtered between 0.1 and 100 Hz to remove slow drifts over time.

Digital triggers were sent by means of a VPixx Technologies (Saint-Bruno, Canada) screen.

## EEG preprocessing and statistical analysis

### Preprocessing

Preprocessing was carried out in Letswave 5 (Mouraux & Iannetti, 2008), an open-source toolbox running in MATLAB 2016b.

First, the EEG signal was filtered with a bandpass fourth-order zero-phase Butterworth filter between 0.1 and 100 Hz and downsampled to 256 Hz. Subsequently, data were segmented in epochs starting 2 s before stimulation onset until 2 s after stimulation offset. Independent component analysis was computed using a square mixing matrix algorithm, and the component that more strongly related to eye blinks was removed in each subject (components were identified through their topography and the correspondence between their time course and that of frontal electrodes located above the eyes). Data were then visually inspected and cleaned using trial substitution and channel interpolation. First, bad trials flagged during data acquisition and therefore repeated at the end of the experiment were compared with their repetition, and the one containing more noise (larger fluctuation or noise over a larger number of channel) was removed. Subsequently, noisy channels were visually identified and replaced through linear interpolation of their three closest neighboring electrodes (max. 5% of all scalp electrodes were interpolated per observer). Data were then re-referenced to the average of all electrodes and additionally cropped between 2 s after and before stimulation onset and offset, respectively. This resulted in segments of approximately 58.33 s (= 14,933 bins). This specific length was selected as it corresponds to an integer number of oddball cycle, which will allow for precise extraction of frequencies of interest. Finally, epochs were averaged together within condition for each subject separately.

### Frequency domain analysis

Frequency spectrum was extracted from averaged epochs by means of fast Fourier transform (FFT), MATLAB’s built-in function.

#### Significant harmonics

In order to assess the number of base and oddball harmonics to include in the response quantification, data were averaged across subjects and 26 occipital temporal electrodes (selected based on previous studies and visualization of the current data set through conditions and subjects; A9–A16, A22–A29, B6–B12, D30–D32). The grand-average signal was *z*-scored (20 surrounding frequencies, excluding immediately adjacent and two most extreme bins [minimum and maximum]), and responses to base and oddball fundamentals and harmonics were isolated. Harmonics were retained for further analysis if their *z*-score exceeded the 1.64 (*p* < 0.05, one-tailed) threshold for more than half of the conditions. This led to four significant harmonics for the base frequency (from 6 to 24 Hz) and 10 harmonics for the oddball frequency (from 0.8572 to 8.572 Hz), excluding the seventh as it corresponds to the fundamental of the base. However, as the 10th harmonic frequency falls within the alpha range, we only retained harmonics up to, and including, the 9th (i.e., 7.715 Hz).

#### Response quantification

In order to quantify base and oddball responses, FFT signals were first baseline corrected by subtracting from each frequency the average of 20 surrounding frequencies (excluding immediately adjacent and two most extreme bins [minimum and maximum]). Subsequently, baseline-subtracted harmonics were summed together.

### Relationship between base and oddball responses

Visual inspection of the data suggested that both the base and the oddball response amplitude increased at larger sizes. We performed within-subject Spearman correlation to explore whether the two responses covaried. We applied Holm–Bonferroni correction to control for multiple comparisons.

### Effect of stimulus size on response amplitude

To evaluate the impact of stimulus size on both the base and oddball responses amplitude, 26 occipitotemporal electrodes were pooled together (i.e., A9–A16, A22–A29, B6–B12, D30–D32). Subsequently, size effect was evaluated for each VP separately using a linear mixed model. To account for interindividual variations, the variable subject was added to the model, which can be summarized as follows:

(1) $$\begin{array}{c} \mathrm{Amplitude}∼\mathrm{Size}+\left(1|\mathrm{Subjects}\right).\; \end{array}$$

Post hoc contrasts between each size were corrected for multiple comparisons using Tukey's honestly significant difference (HSD) test.

### Significance and outlier detection

The impact of size and viewing position variation was evaluated based on two criteria. The first is response significance. The main goal was to determine whether a response can be reliably obtained from a large portion of the subjects independently of condition, or on the contrary, some parameters are suboptimal for such purpose.

Second, we aimed to determine the extent and homogeneity of VP-dependent preference across sizes. Specifically, our goal was to assess whether at some sizes, the neural bias would either decrease or converge across subjects toward the same VP.

#### Response significance detection

We first determined response significance at the individual level. We reasoned that if a size would systematically fail to elicit a significant response, it could be excluded from further statistical analysis on VP-related preference, hence reducing data dimension and number of comparisons.

Therefore, for each type of response (base and oddball), each subject, and each condition independently, FFT epochs were cropped into segments composed of group-significant harmonics (first to fourth for base response and first to ninth harmonics, excluding the seventh for the oddball) and 24 surrounding bins. These smaller epochs were then summed together before pooling 26 occipital temporal electrodes together. Finally, the *z*-score of the frequency of interest was computed by means of the same parameters used at the group level.

Responses above 1.64 (*p* < 0.05) were considered significant. In order to later compute proportions, responses were then relabeled as 1 if they were significant and as 0 otherwise. However, the focus of subsequent analyses was more on the *absence* of a significant response. Therefore, to facilitate visualization, proportion, and proportion difference estimation of nonsignificant responses, binomial values were switched, with 1 indexing a nonsignificant response and 0 a significant one.

#### Outlier detection

Within this article, we defined a preferential response as a significantly stronger, or outlier, response for a VP compared to responses to other VPs. Therefore, outlier detection was performed on each subject and size independently by estimating the deviation of each VP-related response through the median absolute deviation (i.e., MAD). Therefore, each response was converted into its deviation (MAD_score_) as follows:

(2) $$\begin{array}{c} \mathrm{MA}D_{\mathrm{score}}=\left(x_{i}-M_{j}\right)/\mathrm{MAD},\; \end{array}$$

where

$$\begin{array}{c} \mathrm{MAD}=c*\mathrm{median}(|x_{i}-M_{j})|), \end{array}$$

where *x_i_* is the *i*th item of the data series, *M_j_* is the median of the data series, and *c* = 1/0.75 quantile of the data series.

Any response with a deviation exceeding 2.5 was considered an outlier (Leys et al., 2013). As for significance detection, responses’ deviation score was relabeled as 1 if the response was considered an outlier and 0 otherwise. Importantly, subjects might exhibit more than one outlier response. To avoid considering each subject multiple times, for statistical comparison of the total number of subjects exhibiting outlier responses, each participant was counted not more than once.

Complete information regarding the number of subjects showing outlier or nonsignificant responses across VPs and size was then summarized separately.

Changes in proportion in terms of nonsignificant or outlier responses were evaluated by means of the McNemar mid-*p* test, which is well suited to compare dependent samples of medium or small sizes (Fagerland et al., 2013). Multiple comparisons were controlled using Holm–Bonferroni correction. Moreover, 95% confidence intervals of proportions and proportion differences were estimated by means of 5,000 bootstrap samples.

Finally, after identifying the condition(s) most optimal in terms of significant responses and outlier convergence, we explored the impact of these conditions on subjects for whom it did not trigger extremely large responses. Our aim was to identify not only the condition most optimal for the majority of subjects but also the one that was the least suboptimal (i.e., triggering weaker within-subject responses compared to other conditions). To do this, for each size of interest, we explored the response triggered by the “optimal” VP in terms of significance and how it ranked with respect to responses evoked by other viewing positions.

## Results

### Base response

The neural response at the general frequency was computed to ensure the proper synchronization of the visual system to the visual stimulation.

**Figure 3.**
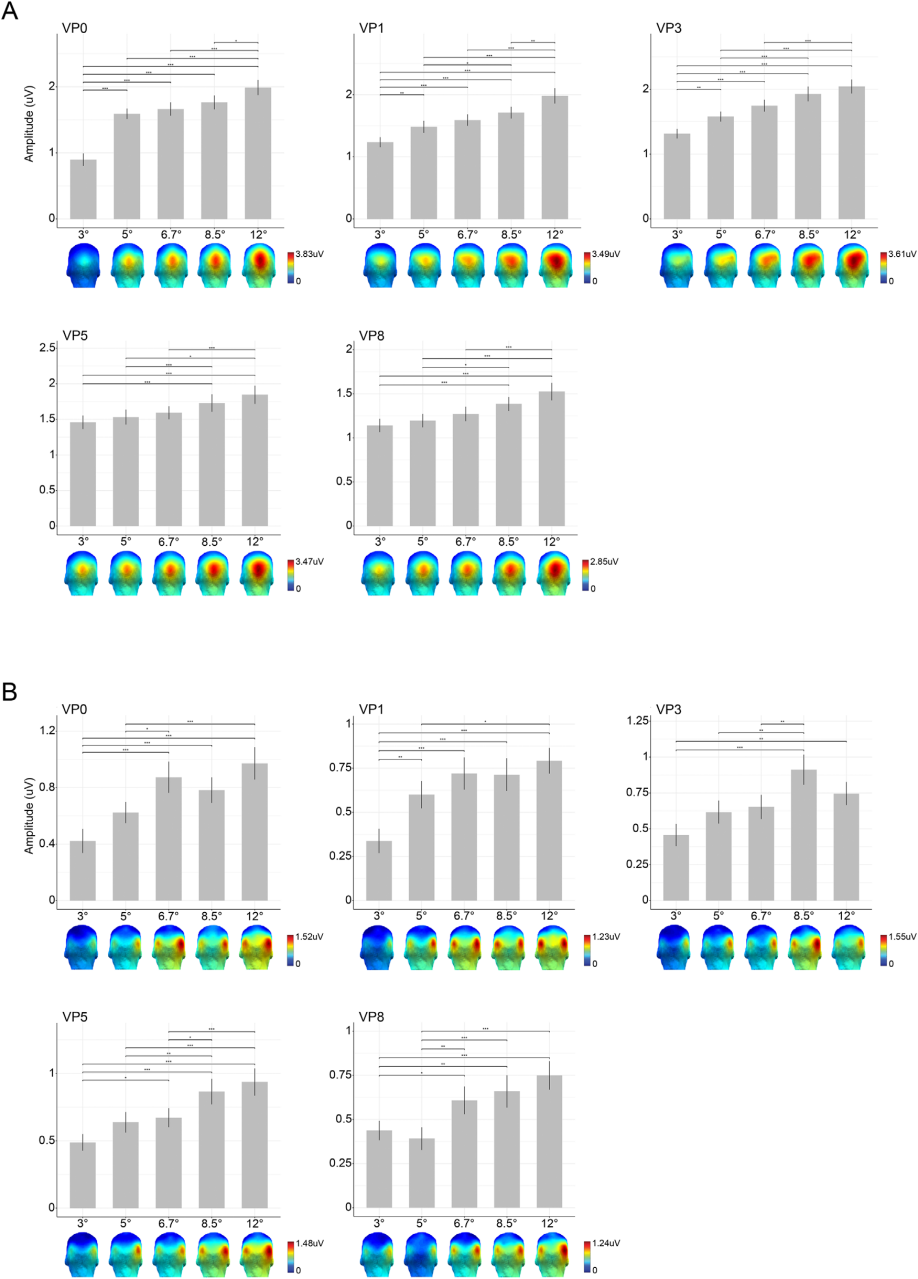
Grand-average base and oddball responses for all sizes and viewing positions. Responses at the base (A) and oddball (B) frequencies are visualized as the average across subjects and as the average over occipitotemporal electrodes (bars) or at each electrode (topographies). Error bars represent the standard error. *p < 0.05. **p < 0.01. ***p < 0.001.

The results of the linear mixed model evaluating the effect of size on the response amplitude show that size has a significant effect on response amplitude at all VPs: VP0, *F*(4, 136) = 56.33, *p* < 0.0001; VP1, *F*(4, 136) = 30.11, *p* < 0.0001; VP3, *F*(4, 136) = 33.84, *p* < 0.0001; VP5, *F*(4, 136) = 9.96, *p* < 0.0001; and VP8, *F*(4, 136) = 13.42, *p* < 0.0001. Post hoc comparisons showed that the base response grows significantly as stimuli's size increments (Figure 3A). Topography shows a medial-occipital distribution for conditions where fixations were directed toward the center of the face. When fixations were enforced on the left and right eyes, we found an additional activity in ipsilateral occipital regions, which increases with stimuli's size (Figure 3A).

At the individual level, the base response was significantly stronger than surrounding noise in all 25 conditions for all 35 subjects except for 1 condition (i.e., 3°-VP0) for S16.

### Oddball response

#### Group level

The oddball response was used as an index of neural FID. The results from the linear mixed model revealed a main effect of stimulus size on response amplitude, independently of the VP considered: VP0, *F*(4, 136) =14.21, *p* < 0.0001; VP1, *F*(4, 136) =13.77, *p* < 0.0001; VP3, *F*(4, 136) = 9.40, *p* < 0.0001; VP5, *F*(4, 136) = 14.83, *p* < 0.0001; and VP8, *F*(4, 136) = 11.64, *p* < 0.0001. Post hoc contrast between sizes showed that response amplitude increases significantly with stimuli's size. However, it plateaus at size 6.7° or 8.5° depending on the VP considered, in which case a further size increment does not lead to a significantly larger response. Figure 3B summarizes the group responses as well as the results of the contrasts extracted from the linear mixed model.

#### Individual level

##### Base and oddball relationship

**Figure 4.**
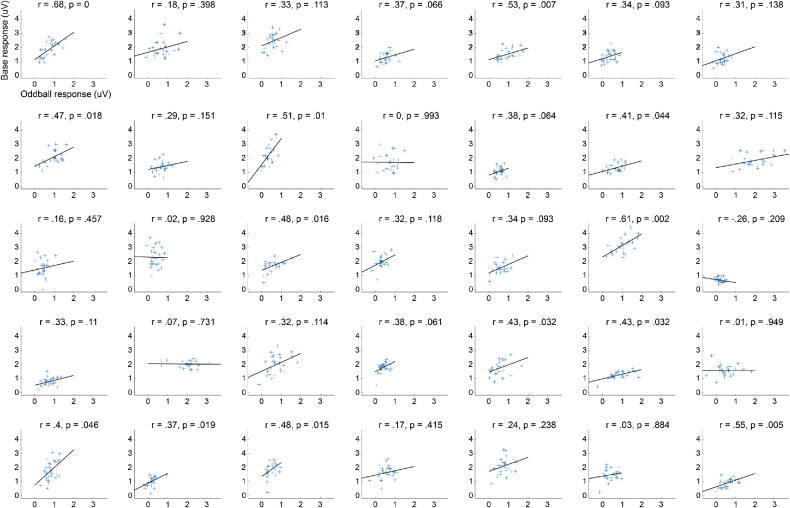
Relationship between base and oddball responses. Scatterplot of the base (y-axis) and oddball (x-axis) responses for all condition for each subject separately. Correlation coefficient and *p* value are reported for every observer.

Holm–Bonferroni corrected Spearman correlations between oddball and base response resulted in only one subject showing a significant relationship between responses at the base and oddball frequencies (S1, *r* = .68, *p* < 0.001; see Figure 4 for all subjects).

##### Response significance

**Figure 5.**
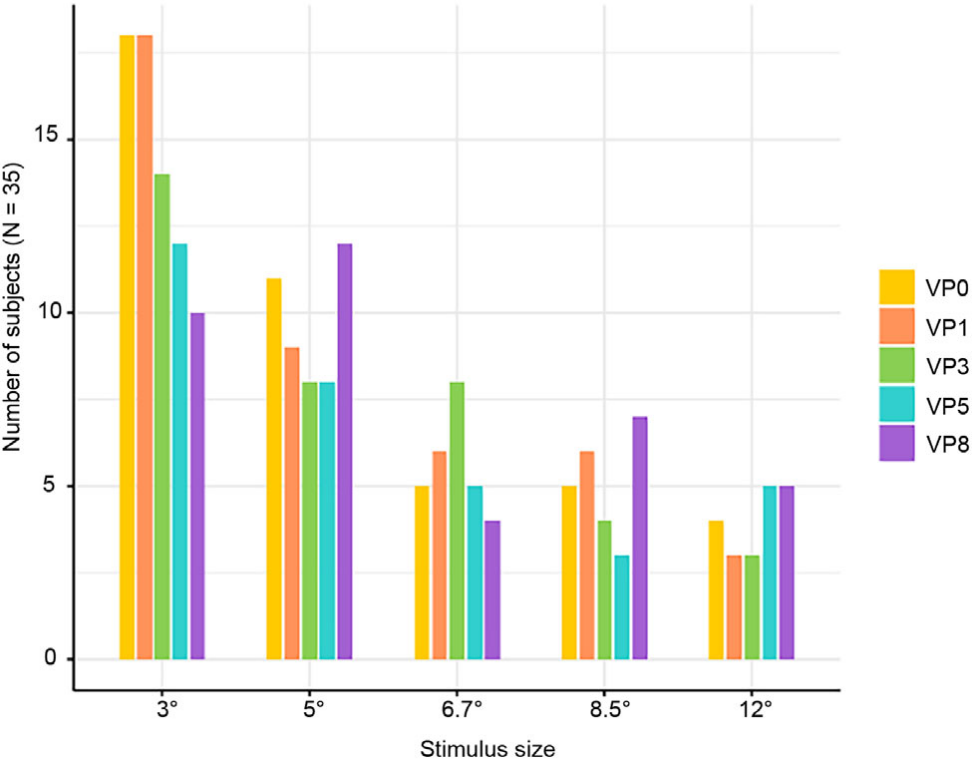
Distribution of nonsignificant (NS) responses across size and VP in terms of number of subjects. The same subject could have more than one NS condition and therefore the cumulative sum within one size or VP can exceed the total number of subjects (*N* = 35).

**Figure 6.**
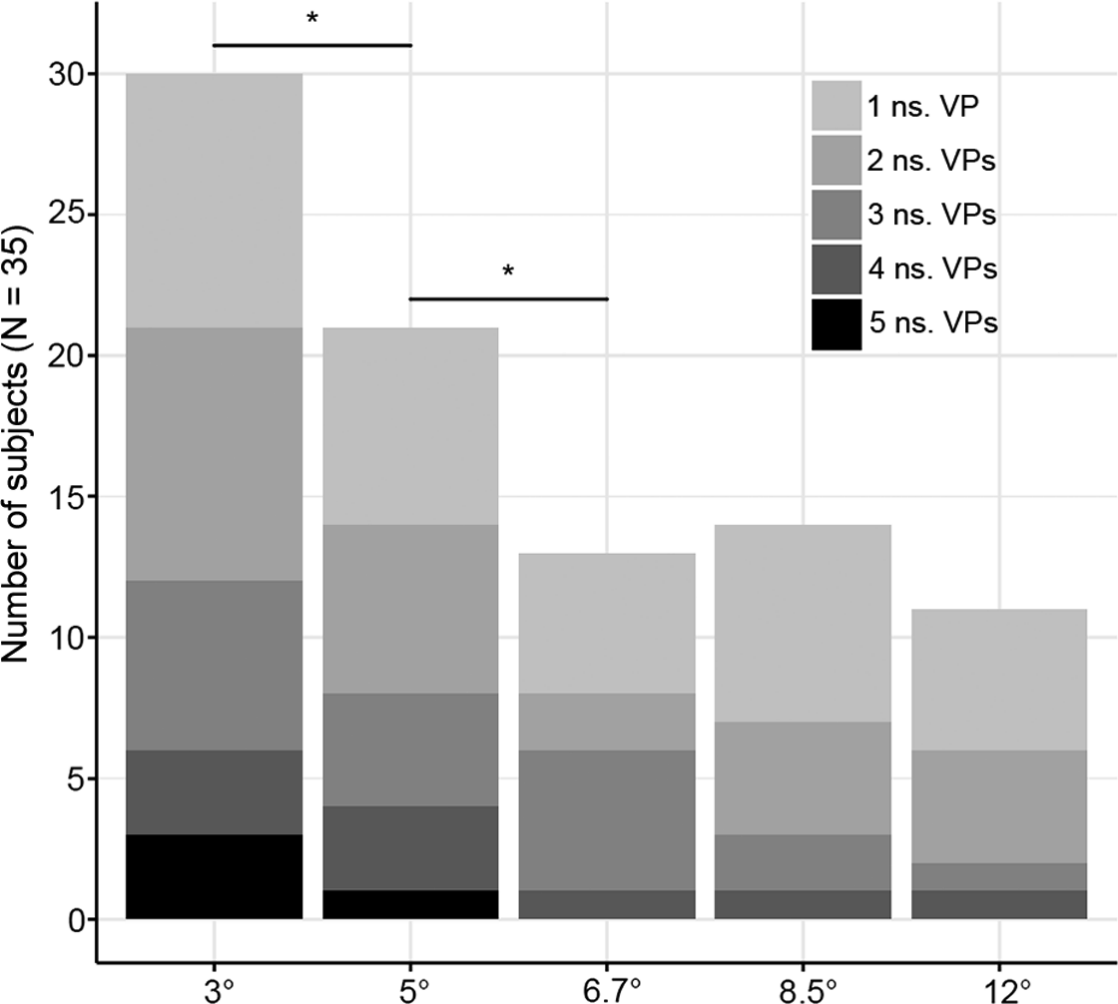
Number of subjects showing a nonsignificant response according to only stimulus size. Stack bars illustrate the exact number of VPs that were NS for each subject. For example, green bars represent the number of subjects with three nonsignificant responses in each size. *Significant differences between the number of subjects at mid *p* < 0.05.

The significance of the response was severely modulated by the stimulus size. Figures 5 and 8 highlight the number of subjects who failed to show a significant response according to size and VP. As it becomes apparent, the smallest size that was tested was associated with the largest number of nonsignificant (NS) responses. Importantly, most subjects (i.e., 27/35) exhibited nonsignificant responses for some viewing positions but significant neural FID for other VPs (Figures 6 and 8, Supplementary Table S1). To ensure that significance of those responses was not due to a subtle difference, significant responses were also evaluated against a more severe threshold of *p* = 0.01. This showed that a pattern of significant (at *p* = 0.01) and nonsignificant (at *p* = 0.05) persisted in 25 subjects (Supplementary Table S1 for *z*-score of all subjects in all conditions).

Collapsing all VPs together within each size shows that at the smallest stimulus size (3°), 85.7% (95% confidence interval [CI]: 74.4, 97.1) of subjects exhibited at least one nonsignificant response. When considering the next size (5°), the total number of NS subjects decreased by 9 (25.7%; 95% CI: 9, 42.5; mid *p* = 0.0032), leaving only 60% (95% CI: 43.4, 76.6) of subjects with at least one nonsignificant response. A similar significant decrease (8 subjects, 22.9%; 95% CI: 8.7, 37.1; mid *p* = 0.0018) was observed between sizes 5° and 6.7°, where less than half of the subjects (37.1%; 95% CI: 20.8, 53.5) exhibited a nonsignificant response (Table 1, Figures 5 and 8). As the size kept increasing, the number of nonsignificant subjects stabilized, and the remaining differences were no longer significant (see Figure 6 for all differences).

**Table 1.** Proportion and proportion difference across conditions of number of subjects exhibiting a non-significant neural FID response.


Proportion (%)				
3°	5°	6.7°	8.5°	12°
85.7 (74.4, 97.1)	60.0 (43.4, 76.6)	37.1 (20.8, 53.5)	40.0 (23.7, 56.3)	31.4 (16.1, 46.8)
Proportion difference between groups (%)				
3°–5°	5°–6.7°	6.7°–8.5°	8.5°–12°	
25.7 (9.0, 42.5)	22.9 (8.7, 37.1)	2.9 (−5.6, 11.6)	8.6 (−2.8, 19.9)	

*Note.* Number in parentheses are 95% confidence interval estimated as ± 2 standard errors.

##### Outlier VP-dependent responses

Because the two smallest sizes (i.e., 3° and 5°) triggered a nonsignificant response in more than half of the subjects, we decided to not investigate these conditions beyond descriptive statistics and to mainly focus on the remaining sizes.

**Figure 7.**
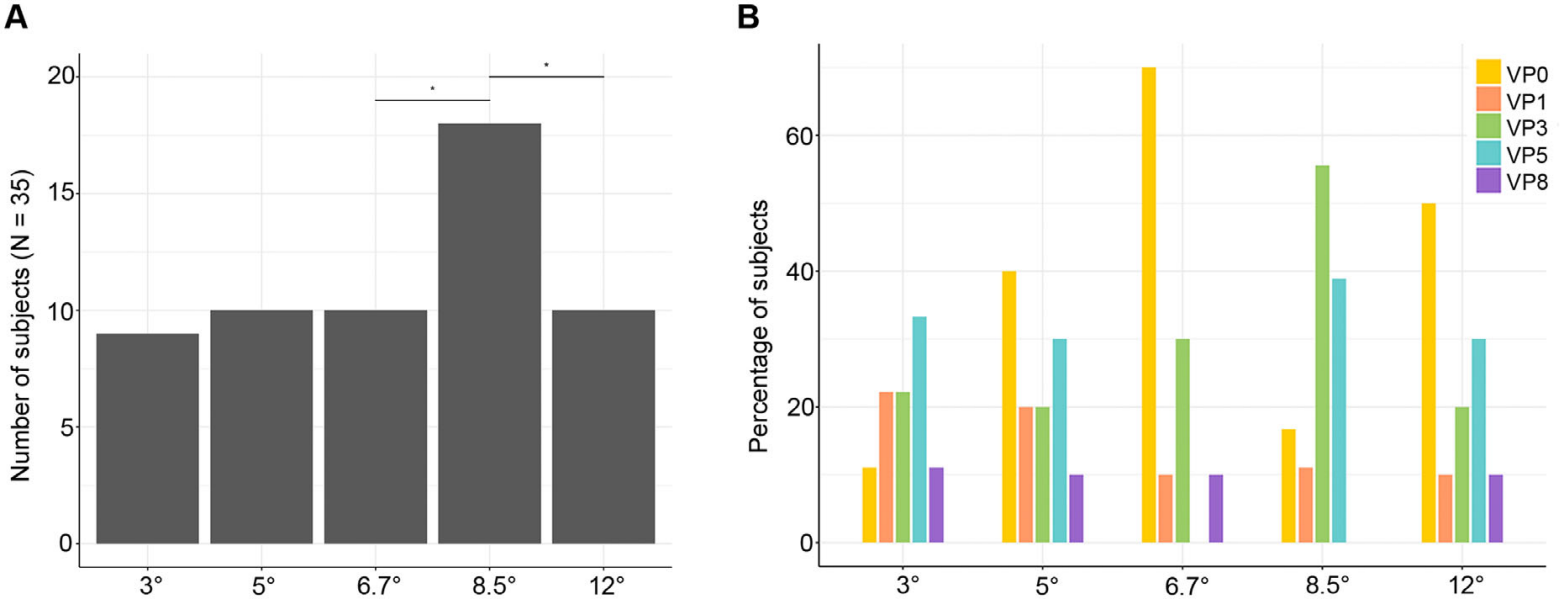
Distribution of outlier response. (A) Number of subjects showing at least one outlier response. *Significant difference in total number of subjects at mid *p* < 0.05. (B) Distribution of subjects exhibiting outlier responses across VPs and sizes. The y-axis represents the number of subjects showing an outlier response for a given VP over the absolute number of subjects showing at least one outlier response at a given size. Please note that a subject could exhibit more than one outlier response per size, and as such, numbers do not perfectly add up in A and could lead to percentages more than 100%.

As shown in Figure 7A, size 8.7° led to more than half of the subjects to show at least one outlier response (51.4%; 95% CI: 33.5, 69.3), while in size 6.7° and 12°, this occurred in less than a third of the participants (Table 2). Additionally, the paired difference of eight subjects (22.9%; 95% CI: −1.6, 47.3) between size 8.5° and 12° was significant (mid *p* = 0.039), as well as the paired difference of eight subjects (22.9%; 95% CI: 2.7, 43.1) between size 6.7° and 8.5° (mid *p* = 0.0245) (Table 2).

**Table 2.** Proportion and proportion differences between conditions of number of subjects exhibiting outlier responses.


Proportion (%)				
3°	5°	6.7°	8.5°	12°
25.7 (10.9, 40.5)	28.6 (13.1, 44.0)	28.6 (13.5, 43.6)	51.4 (33.5, 69.3)	28.6 (13.6, 43.5)
Proportion difference between groups (%)				
3°–5°	5°–6.7°	6.7°–8.5°	8.5°–12°	
2.9 (−17.0, 22.7)	0 (−19.4, 19.4)	−22.9 (−2.7, −43.1)	22.9 (−1.6, 47.3)	

*Note.* Numbers in parentheses are 95% confidence interval estimated as ± 2 standard errors.

The outlier distribution across VPs shows that VP0 led to a larger number of outliers in size 6.7° and 12°, while this was obtained by VP3 in size 8.5°. Importantly, VP0 in size 6.7° was the outlier response for 70% of the subjects showing an extreme response. On the other hand, VP0 included only 50% of subjects in size 12° and VP3 55.6% of subjects in size 8.5° (Figure 7B).

**Figure 8.**
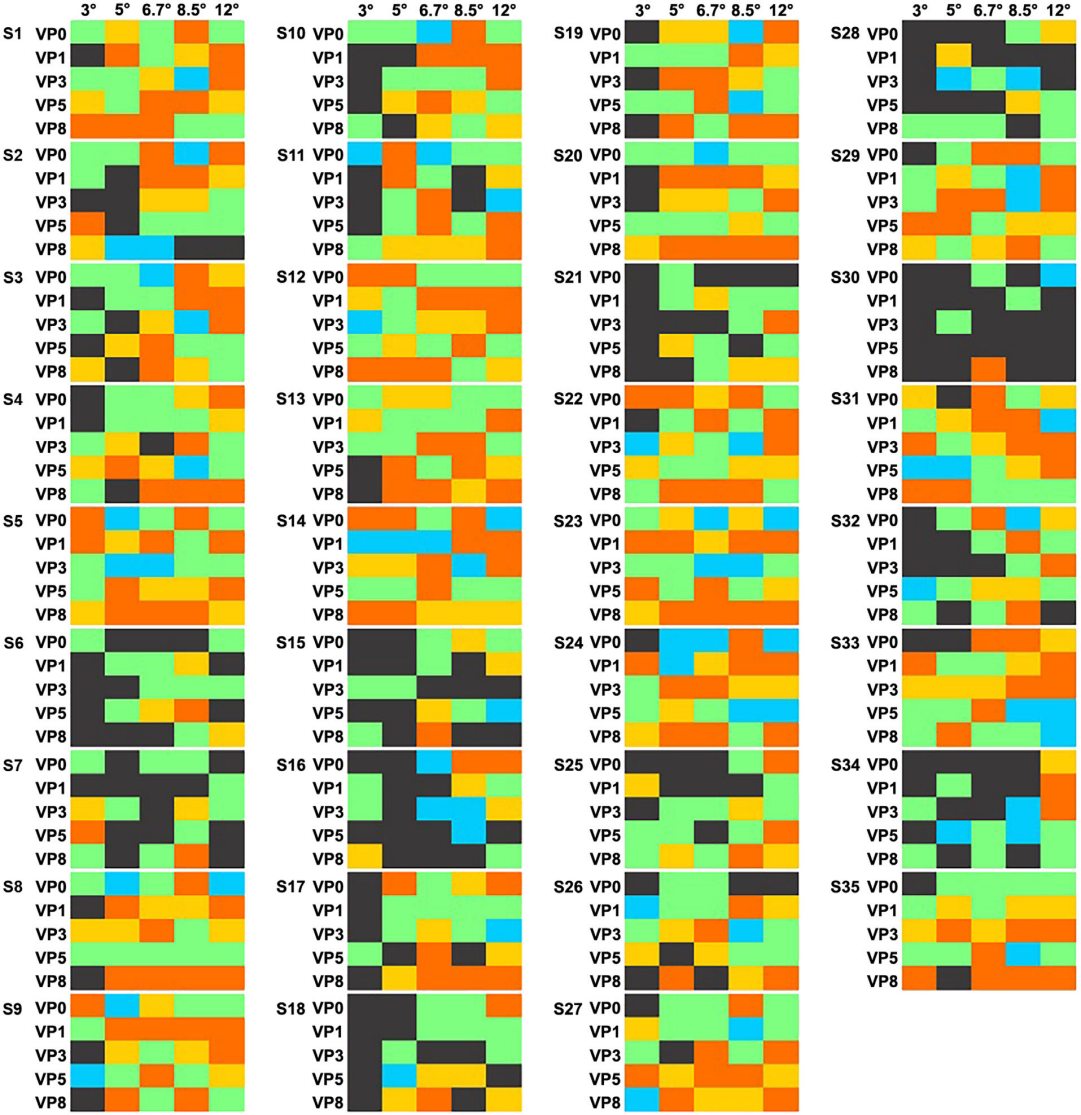
Summary of each subject's MAD score and response significance. If a response *z*-score was below 1.64 (*p* < 0.05), it was color coded as dark gray. Significant responses were color coded according to their MAD score. Orange indicates a MAD score < 0, yellow indicates a MAD score = 0, light green indicates a MAD score > 0, and light blue indicates a MAD score > 2.5 (outlier).

Finally, at size 6.7°, 8.5°, and 12°, fixation on VP0, VP3, and VP0, respectively, led to a nonsignificant response in five, four, and four subjects (Figure 5). Excluding those, the same viewing positions also triggered a response amplitude below the median (i.e., MAD_score_ < 0; with respect to other viewing positions within the same size) in five, five, and six additional subjects (Table 3, Figure 8).

**Table 3.** Number of subjects exhibiting a MAD score above, equal to, or below different thresholds for each VPs and Sizes.

	MAD > 2.5	MAD > 0	MAD = 0	MAD < 0
3°				
VP0	1	10	1	5
VP1	2	7	4	4
VP3	2	13	5	1
VP5	3	11	4	5
VP8	1	11	8	5
5°				
VP0	4	11	4	5
VP1	2	13	5	6
VP3	2	13	8	4
VP5	3	14	6	4
VP8	1	2	4	16
6.7°				
VP0	7	14	4	5
VP1	1	16	4	8
VP3	3	6	9	9
VP5	0	11	7	12
VP8	1	10	5	15
8.5°				
VP0	3	11	4	12
VP1	2	8	6	13
VP3	10	9	7	5
VP5	7	11	9	5
VP8	0	7	7	14
12°				
VP0	5	14	6	6
VP1	1	8	8	15
VP3	2	11	3	16
VP5	3	15	8	4
VP8	1	9	8	12

*Note.* Subjects were included in these categories only if they exhibited a significant neural FID.

## Discussion

This study aimed to assess the impact of stimulus size on rapid neural FID response as a function of the fixated face region (VP). We tested participants with fast periodic visual stimulation in which a stream of same-identity faces was presented at a general base frequency of 6 Hz, with different-identity faces periodically interleaved every seventh image (i.e., 0.85 Hz) in the sequence. Across conditions, stimuli varied in their size and in the VP that participants were required to fixate.

### The impact of face size on neural face discrimination responses

Our data show that stimulus size modulates the amplitude of neural FID: The smaller the stimuli, the weaker the response. Interestingly, while amplitude appears to linearly increase across stimulus sizes at the base response, the oddball response seems to benefit from stimulus size increments only until 6.7° to 8.5° of visual angle but does not show significant amplitude increases for 12°. This observation could be explained by the modulations of the visual processes involved in the extraction of information used during FID. Specifically, identity processing involves both a holistic coarse percept of faces and a finer-grained analysis of information that are idiosyncratic to each face (e.g., Rossion, 2008; Rossion et al., 2011). At small sizes (e.g., 3° of visual angle), the rapid extraction of detailed idiosyncratic information might be more difficult, especially given that stimuli remained on screen for only 166 ms, allowing for only one fixation at a predetermined location. As stimulus size grows and visibility increases, this analysis of fine components and their integration into a whole might become easier.

The pattern of neural response amplitude as a function of size mimics the pattern of face recognition behavioral performance reported by Yang et al. (2014), in which stimulus size was also parametrically manipulated. In this later study, performance improved from 1° to 7° of horizontal visual angle, reaching a plateau thereafter. Yang et al. (2014) suggested that the improvement might relate to the involvement of holistic processing occurring for faces larger than 6° of horizontal visual angle. However, the question of why such an increase levels off remains unanswered. In opposition to Yang et al. (2014), McKone (2009) reported that holistic processing during face categorization is impoverished for stimuli larger than approximately 6.5° of vertical visual angle. However, one must emphasize that McKone (2009) used different stimuli (i.e., Mooney faces) and a different task (i.e., face categorization/detection). As such McKone's (2009) observations and theoretical implications might not necessarily apply to face identity discrimination. However, the mechanisms behind these observations remain open for discussion.

While the cutoff for optimal holistic processing might not be the same across different face subprocesses, it would be interesting to investigate whether, as for categorization, also in identity processing there is a size limit beyond which holistic processing would be impaired. Regardless of this theoretical debate, further studies are required to explore the question of whether neural FID decreases for stimuli larger than 12° of vertical visual angle (which relates to the plateau observed here).

### The impact of face size on VP-dependent neural FID

The main goal of the current study was to determine whether reducing face size would reduce neural response variation across viewing positions and/or across observers. We addressed these hypotheses in two steps. First, we assessed the significance of neural responses, so as to exclude from further analysis conditions that were less likely to trigger significant neural FID responses. At the individual level, we observed a greater number of nonsignificant responses for smaller sizes compared to larger stimuli. Additionally, analysis revealed that the number of subjects showing nonsignificant FID responses significantly decreased between 3° and 6.7° of visual angle but did not change substantially between the three largest sizes. Based on these results, we did not further investigate 3° and 5° sizes, as they failed to trigger a systematic neural FID.

Analysis of the data from the three remaining sizes (6.7°, 8.5°, and 12° of visual angle) revealed that the 8.5° size triggered extremely high responses (outlier responses/visual sampling preference) in more subjects than for sizes 6.7° and 12° of visual angle, respectively. Importantly, compared to size 12°, size 6.7° led to a greater agreement between observers. Specifically, at size 6.7°, more than half of those exhibiting an outlier response did so when fixating the central viewing position (VP0), and in general, this viewing position triggered an above-the-median response in 21 out of 35 participants and a nonsignificant response in only 5 of them. Finally, fixation below the nasion still triggered an above-the-median response in two out of three observers when considering only subjects showing a strong positive bias for a different viewing position.

Altogether, these observations indicate that the 6.7° size, which is the smallest among those systematically leading to significant neural FID (i.e., 6.7°, 8.5°, and 12°), reduced individual differences in neural bias the most overall. In fact, a large portion of subjects showed a convergence on VP0 in terms of either outlier responses or above-the-median response. This finding is in line with our expectation that although VP-dependent biases do not appear to decrease with increasingly smaller stimulus sizes, they appear to converge toward the same central viewing position (VP0). At this size, it is likely that both coarse- as well as fine-grained mechanisms can be used to process information gathered from a single fixation in order to reach optimal neural FID. Our data are in line with a previous report by Peterson and Eckstein (2013), who implemented a foveated ideal observer to determine the viewing position maximizing the sampling of information relevant in an identity task. They found that the most effective strategy is to direct the first fixation just below the eyes. For midrange-size stimuli, this location might be strategic as it enables perceiving faces as a unit and at the same time sampling facial regions such as the eyes with relatively high resolution (fixation on VP0 at 6.7° of visual angle places the eyes just outside foveal vision; Figure 2).

### Individual differences when viewing small face sizes

In order to identify an experimental condition that reduces the influence of viewing position on neural FID, we applied different criteria. The first aimed to filter the conditions in terms of robustness of the evoked FID neural responses. Consequently, we discarded the two smallest sizes (3° and 5°), as they led to many nonsignificant responses across conditions. Importantly, while these two conditions were no longer interesting with respect to our original goal, the pattern of responses recorded in these two cases become relevant from a theoretical perspective and is worth discussing. We found that even at the smallest visual angle (3°), most of our observers still exhibited responses indicative of both optimal and suboptimal viewing conditions. In other words, more than three-quarters of our participants exhibited nonsignificant neural FID responses for some VPs (despite showing a significant response to the base stimulation) but at the same time highly significant responses when fixation was constrained to other VPs. These observations might indicate that fixation of specific viewing positions can provide an advantage for neural FID even under challenging conditions, such as when fine-grained information might be more difficult to extract. However, future studies are necessary to clarify what drives these individual differences. For example, a question that cannot be addressed within our current methodology is the contribution of different facial information to the recorded neural response. Although fixation was constrained to a specific viewing position, observers still had access to information from the whole face through parafoveal and peripheral vision. As such, and because there is not a straightforward relationship between the information fixated and its use in natural vision (e.g., Caldara et al., 2010), our data cannot disentangle how these different samplings might have been used to achieve neural FID. By addressing this issue, we could also shed light on the possible strategies for changes across sizes and therefore on potential within-subject difference in VP preferences across scales. Observers could find it more advantageous to sample faces from different locations, since across different face sizes, more or less information can be gathered foveally. For example, on very large images, parafoveal information might not be as useful, and it might be more efficient to locate specific features within the foveal visual field rather than attempting to sample the whole face in one glance by fixating the center of the face.

In line with this reasoning, it would also be important to determine whether these preferences persist for even smaller stimuli (provided that they are able to trigger a significant neural FID). At our smallest size, the foveal visual field systematically overlapped across VPs; nonetheless, stimuli were still too large to allow for an identical foveal input across fixations.

Additionally, in the current study, we did not control for eye movements. That is, subjects had to simply perform an orthogonal task consisting of reporting color changes in the fixation cross. The main goal of the current study was to provide insight to future studies on which experimental manipulation would be the most suited for the largest portion of the tested population. Therefore, we reasoned that for our data to be meaningful in such a context, our settings should have matched those that are commonly used in FPVS-EEG studies, which do not typically monitor eye movements (e.g., Liu-Shuang et al., 2014; Retter et al., 2021). Subsequent experiments that specifically focus on information contribution to neural FID should implement a stricter control of eye movements to ensure the precise fixation at a specific viewing position for the whole duration of the visual stimulation.

Despite these limitations, our findings build on previous reports of individual differences and highlight once again both their complexity and the experimenter's difficulties to control for them. This prompts numerous questions, such as what drives idiosyncrasies, or how do they develop? If studies focusing on cross-cultural differences suggest that individual differences in sampling strategies might be determined by the environment (Blais et al. 2008; Caldara, 2017), then they should also investigate the contribution of the physical properties of the visual system, such as potential individual differences in contrast sensitivity across the visual field. If the most advantageous portion of the visual field for face processing varies across observers, then, as already suggested by Peterson and Eckstein (2013), it is reasonable to expect that this might shape sampling strategies to optimize information intake.

Finally, our results support the possibility that the face system shares similar face-processing mechanisms across observers but does not rely on a universal set of representations to achieve face identification. In fact, the face system is tuned to neural and visual idiosyncrasies (see Stacchi, Liu-Shuang, et al., 2019; Stacchi, Ramon, et al., 2019). These observations might have profound implications for models of information selection and representation, as the same computation should be able to reach effective face recognition with a different set of inputs (i.e., idiosyncratic preferred visual features/representations). This is a challenge that computational modeling studies of face recognition should take over in the future. Future models should not only take into consideration the notion that different observers might prefer different facial information to process facial identity (Stacchi, Liu-Shuang, et al., 2019; Stacchi, Ramon, et al., 2019) but also that this information might depend on the image's scale. Stimulus size should be carefully considered in the models, by adapting visual properties of interest as a function of the scale. The present data, along with the growing literature on individual differences (Arizpe et al., 2017; Mehoudar et al., 2014; Peterson & Eckstein, 2013), make it less likely that a model with fixed rules discarding fixation location and stimulus size could provide a representative description of the human face system. On the contrary, our results strongly support the idea that any computational model of face processing should implement *flexible* rules (see Caldara, 2017), which would allow solving the same task by the use of different strategies.

### Should we standardize the viewing position?

The present study aims to provide a guideline to future studies on which viewing position is the least detrimental to the fewest subjects. Our data show that, among the conditions that we tested, fixation just below the nasion on stimuli subtending 6.7° of vertical visual angle is the one that best meets these criteria.

However, it is important to acknowledge that our data do not support the common practice of standardizing stimulus size and certainly not the viewing position. Our results highlight the complexity of abolishing individual differences and VP-related preferences, as these persisted across all stimulus sizes, and they reinforce the idea that standardizing the viewing position will result in observers’ misrepresentations.

Nevertheless, we are fully aware that testing numerous conditions to ensure the inclusion of the optimal viewing position for each observer is not realistic, and experimenters must compromise on this matter. For this reason, we urge researchers to carefully consider the existence of visual idiosyncrasies for face processing when necessary—for example, when attempting to establish a relationship between neural FID and a behavioral measure, as observers would be forced to fixate a predefined location during neural assessment but might be allowed to sample information using their preferred strategy in the behavioral task. This contrast could lead to a mismatch in task difficulty and potentially return a weaker link between measures. Another scenario where VP standardization might be counterproductive is when investigating neural FID at the single subject level and using this measure as an index to categorize observers. In this context, experimenters should be aware that a weak response might be caused by suboptimal testing conditions, and they should complement their findings with further assessments.

## Conclusions

Our data show that FID neural responses increase with stimulus size, reaching a plateau from 6.7° to 8.5° of visual angle onward. Individual differences across VPs were present for *all stimulus* sizes, even for the smallest. However, importantly, when faces are centered below the nasion and subtend 6.7° of visual angle, these individual differences across VPs are significantly reduced. This stimulus size should be used conventionally to reduce the impact of potential individual differences in neural FID response patterns, as it decreases the likelihood of obtaining and interpreting nonrepresentative measures at the single-subject level. Altogether, these findings highlight the need to rigorously control for face stimulus size and viewing position, while positing a benchmark to reduce FID idiosyncratic responses. Overall, they prompt the necessity for further studies to elucidate the mechanisms at the root of individual differences during face processing.

## Supplementary Material

Supplement 1
